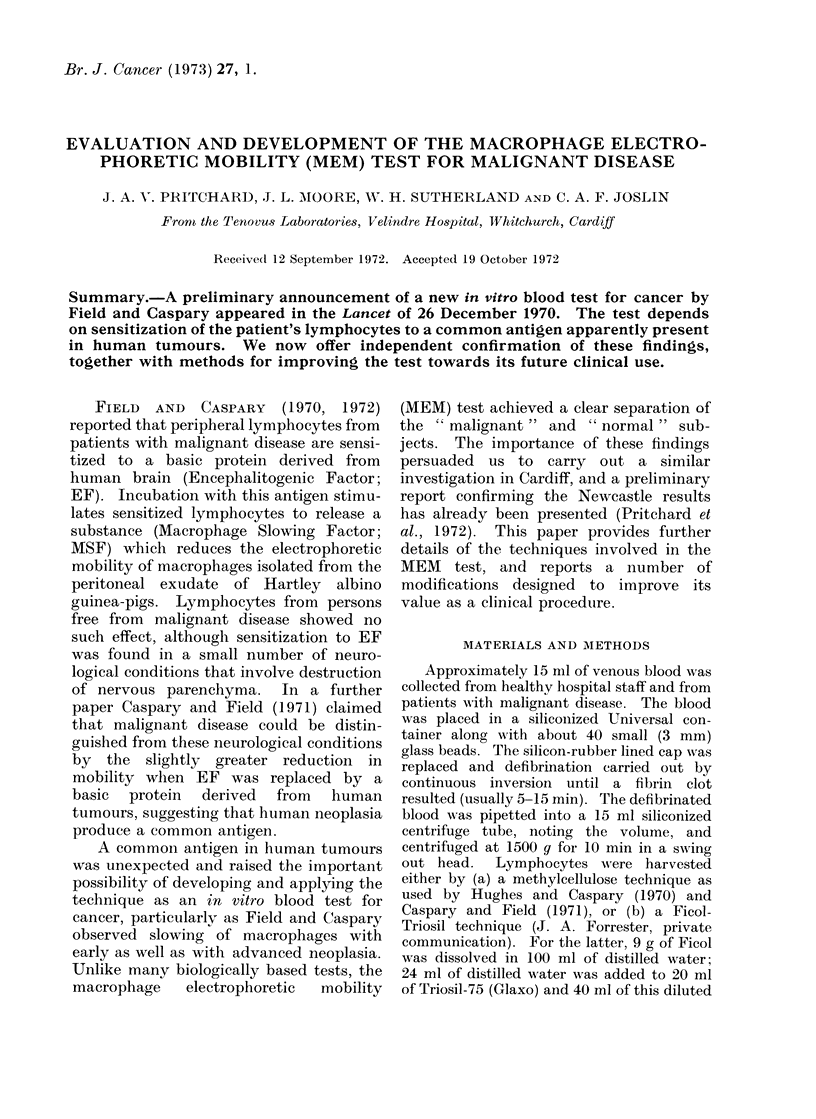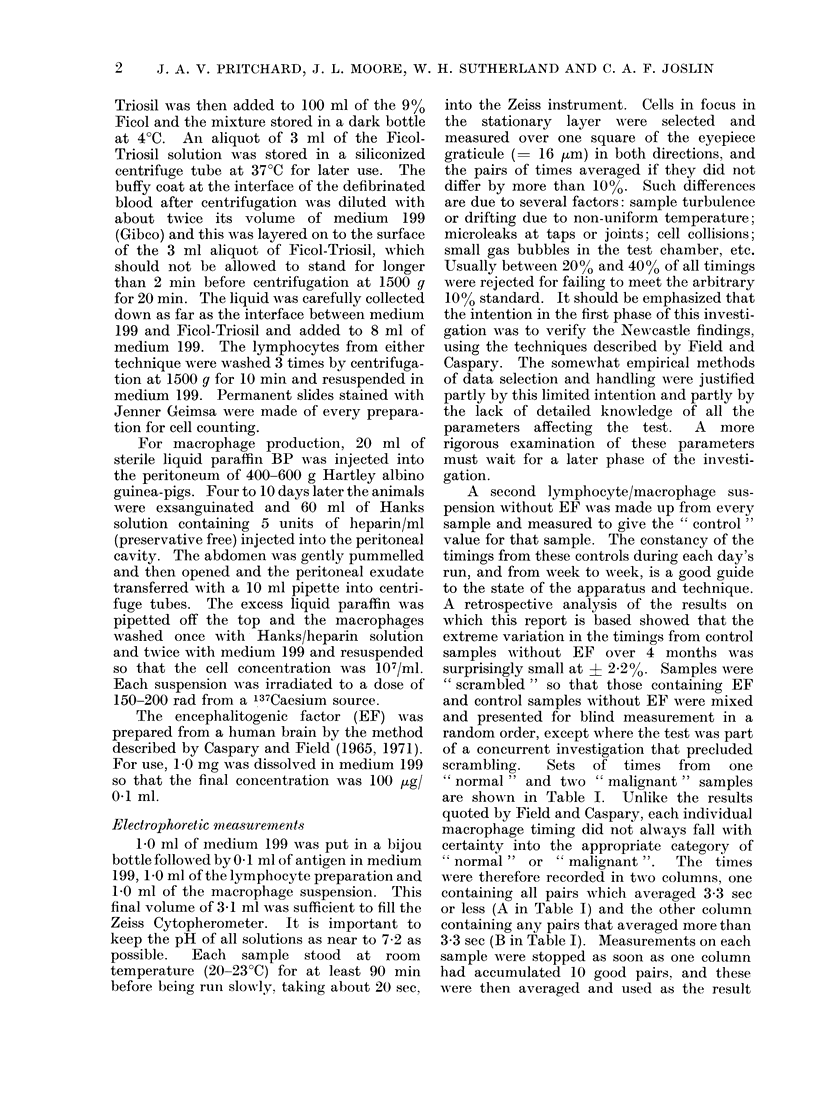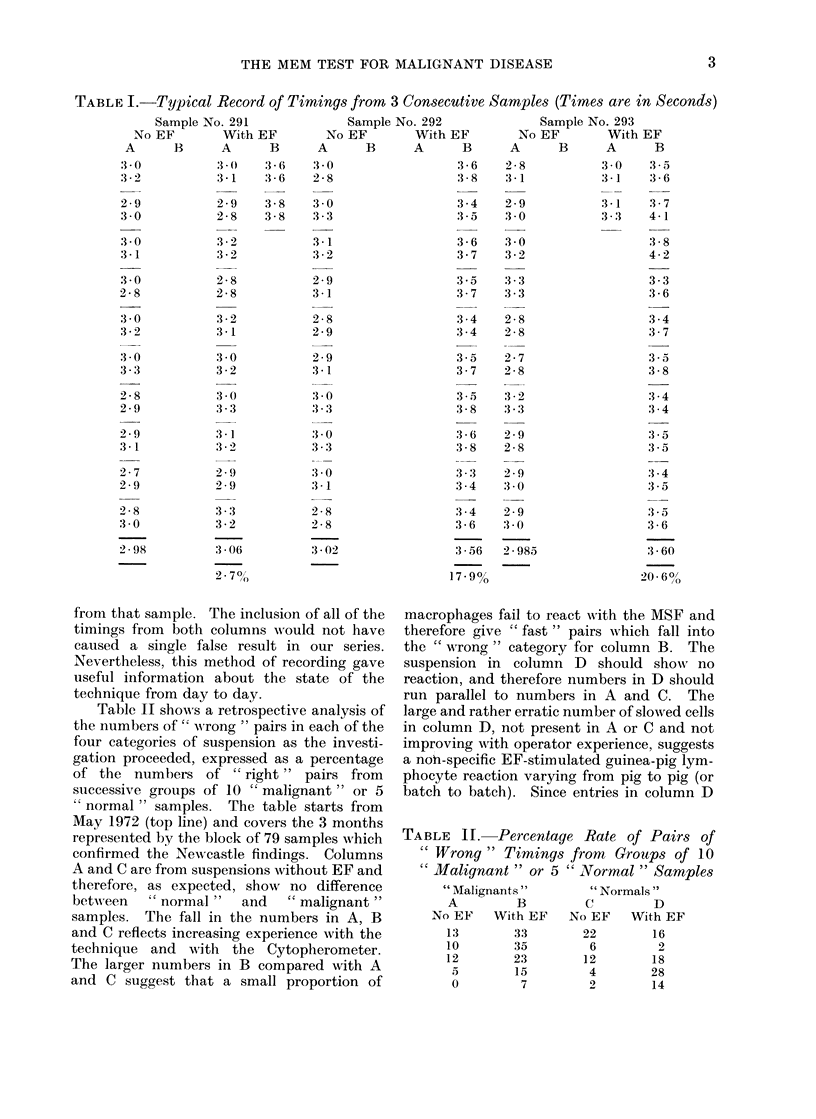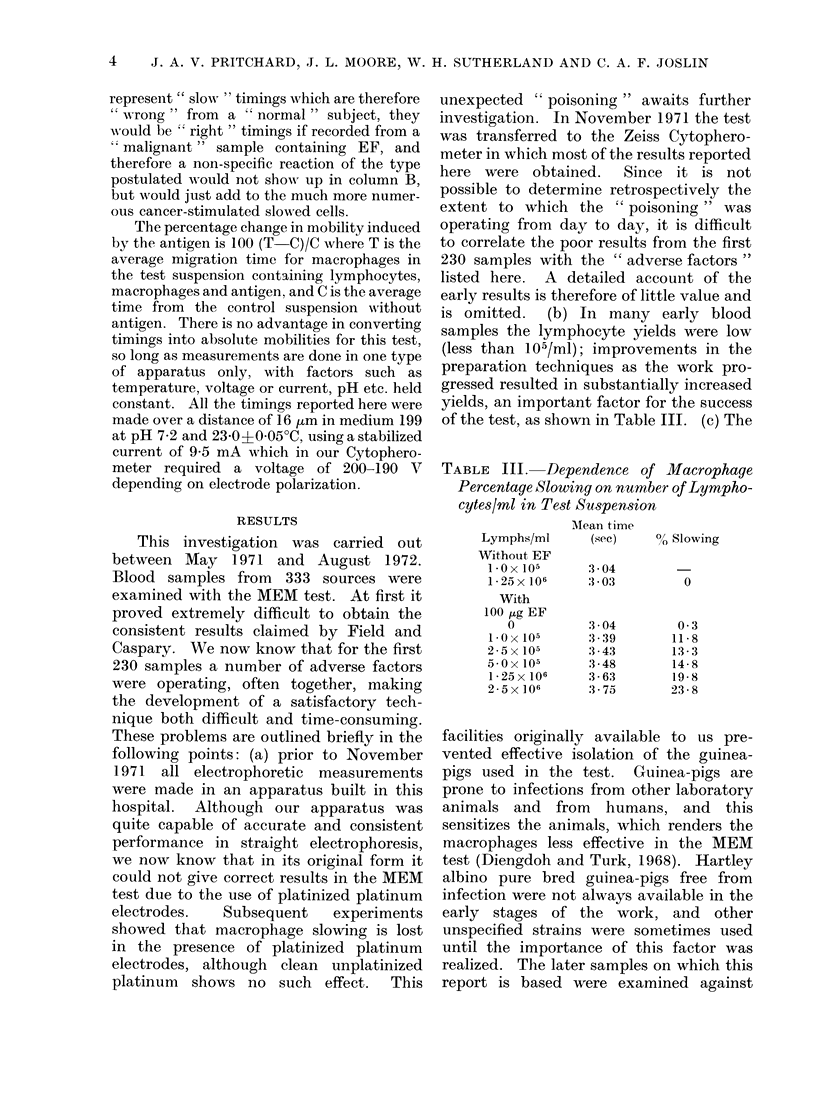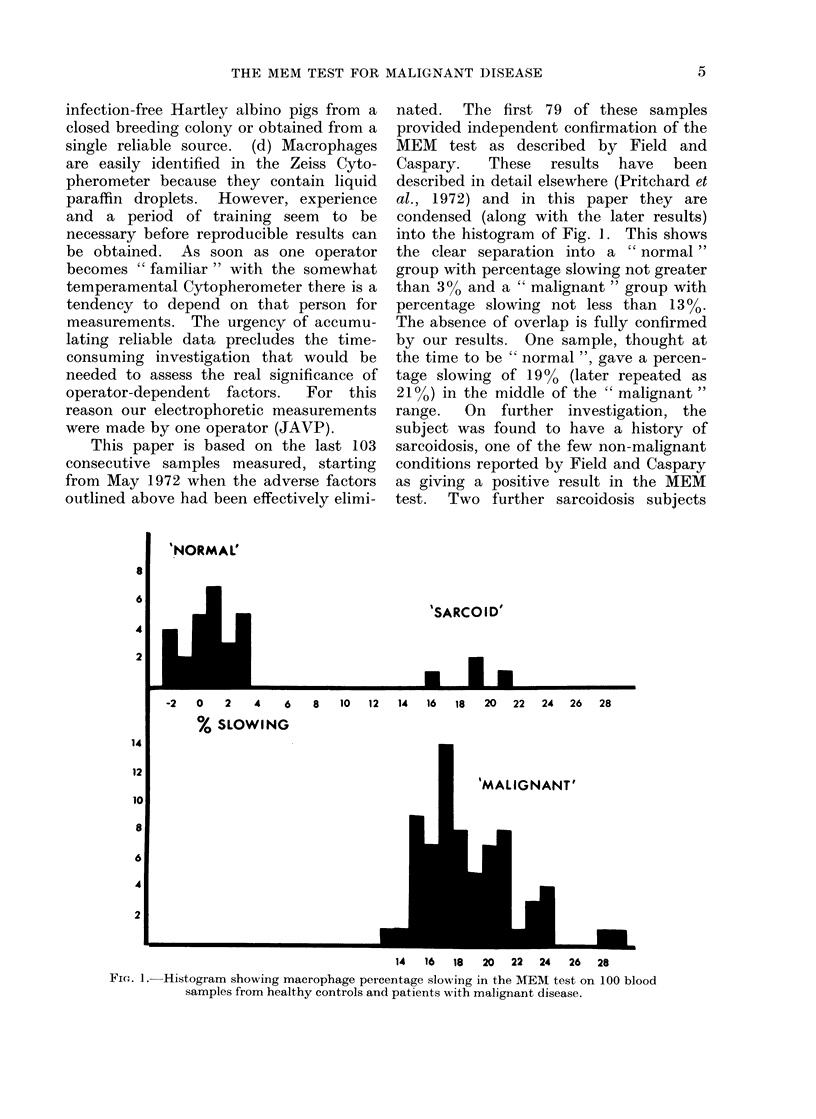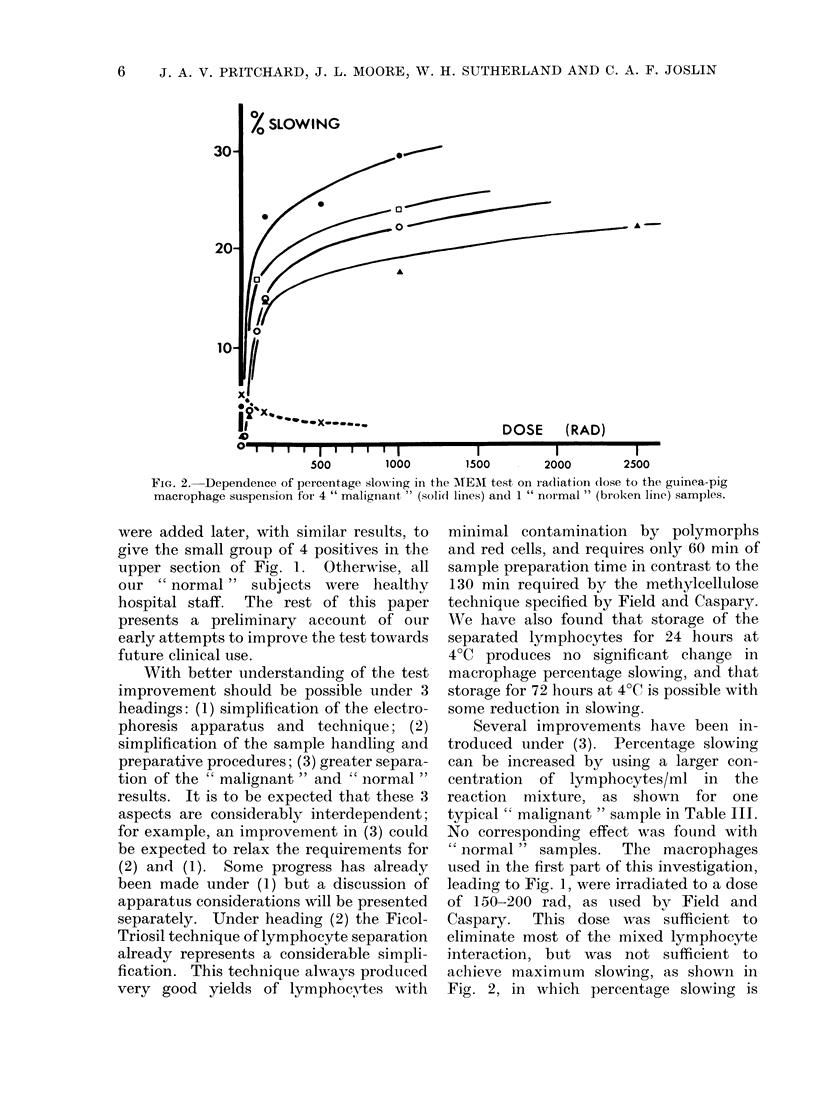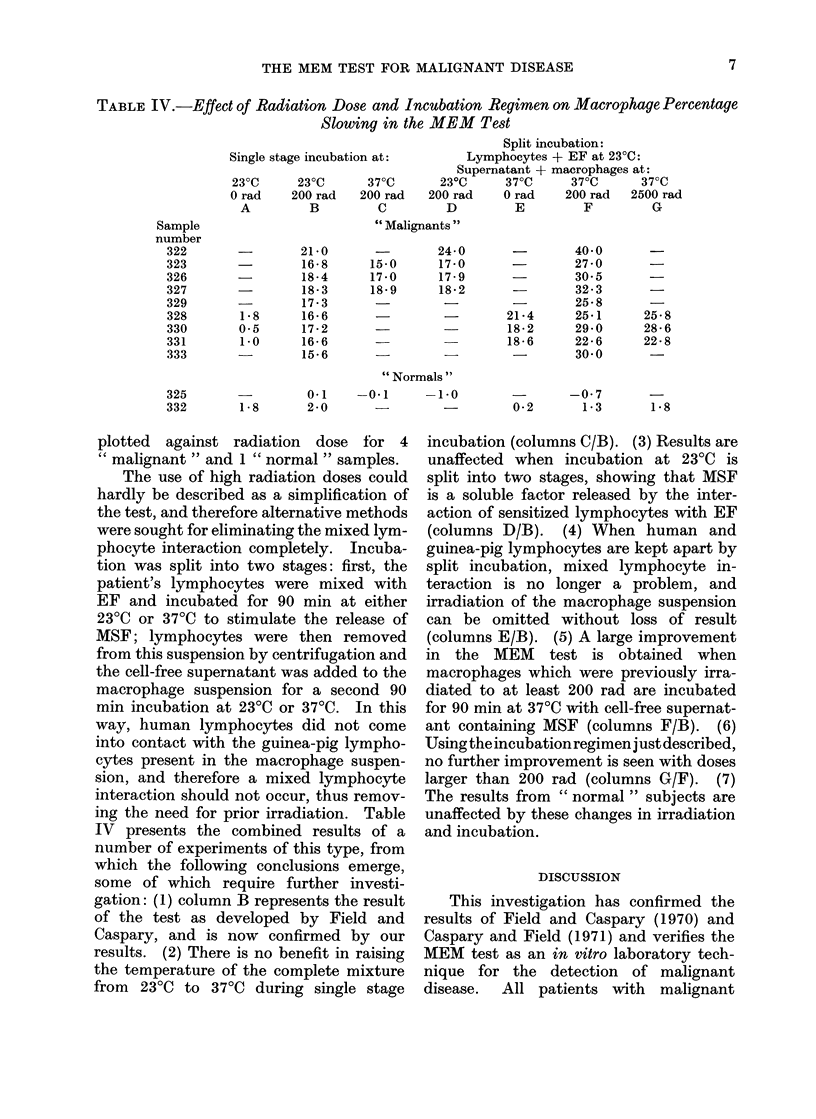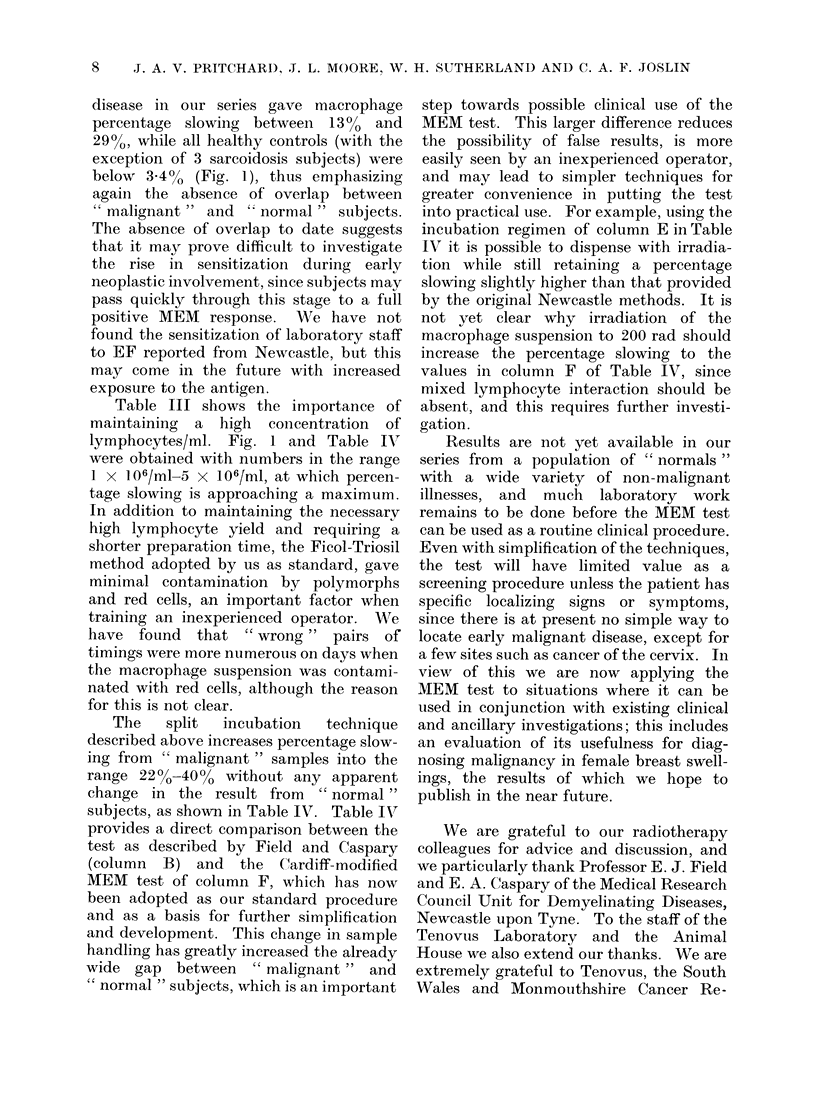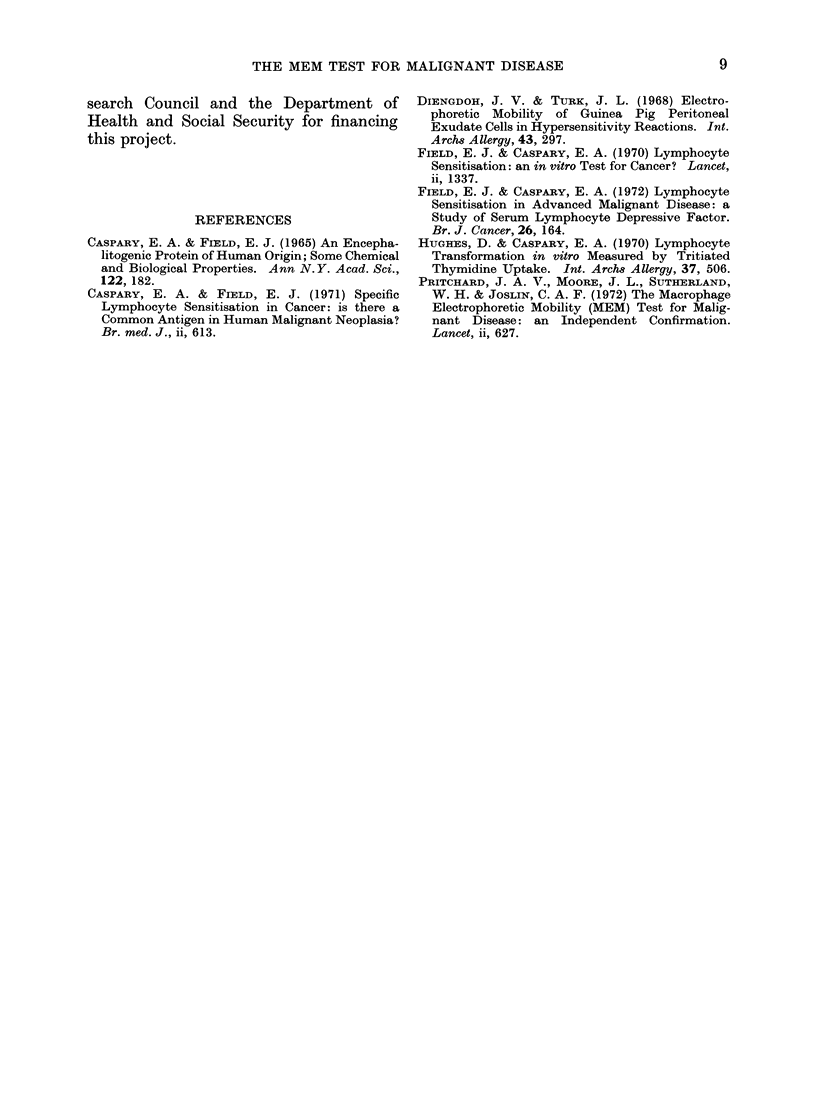# Evaluation and Development of the Macrophage Electrophoretic Mobility (MEM Test for Malignant Disease)

**DOI:** 10.1038/bjc.1973.1

**Published:** 1973-01

**Authors:** J. A. V. Pritchard, J. L. Moore, W. H. Sutherland, C. A. F. Joslin

## Abstract

A preliminary announcement of a new *in vitro* blood test for cancer by Field and Caspary appeared in the *Lancet* of 26 December 1970. The test depends on sensitization of the patient's lymphocytes to a common antigen apparently present in human tumours. We now offer independent confirmation of these findings, together with methods for improving the test towards its future clinical use.


*Br. J. Cancer* (1973) **27**, 1.

### EVALUATION AND DEVELOPMENT OF THE MACROPHAGE ELECTROPHORETIC MOBILITY (MEM) TEST FOR MALIGNANT DISEASE

J. A. V. PRITCHARD, J. L. MOORE, W. H. SUTHERLAND AND C. A. F. JOSLIN

*From the Tenovus Laboratories, Velindre Hospital, Whitchurch, Cardiff*

Received 12 September 1972. Accepted 19 October 1972

**Summary.—A preliminary announcement of a new *in vitro* blood test for cancer by Field and Caspary appeared in the *Lancet* of 26 December 1970. The test depends on sensitization of the patient's lymphocytes to a common antigen apparently present in human tumours. We now offer independent confirmation of these findings, together with methods for improving the test towards its future clinical use.**

FIELD AND CASPARY (1970, 1972) reported that peripheral lymphocytes from patients with malignant disease are sensitized to a basic protein derived from human brain (Encephalitogenic Factor; EF). Incubation with this antigen stimulates sensitized lymphocytes to release a substance (Macrophage Slowing Factor; MSF) which reduces the electrophoretic mobility of macrophages isolated from the peritoneal exudate of Hartley albino guinea-pigs. Lymphocytes from persons free from malignant disease showed no such effect, although sensitization to EF was found in a small number of neurological conditions that involve destruction of nervous parenchyma. In a further paper Caspary and Field (1971) claimed that malignant disease could be distinguished from these neurological conditions by the slightly greater reduction in mobility when EF was replaced by a basic protein derived from human tumours, suggesting that human neoplasia produce a common antigen.

A common antigen in human tumours was unexpected and raised the important possibility of developing and applying the technique as an *in vitro* blood test for cancer, particularly as Field and Caspary observed slowing of macrophages with early as well as with advanced neoplasia. Unlike many biologically based tests, the macrophage electrophoretic mobility (MEM) test achieved a clear separation of the " malignant " and " normal " subjects. The importance of these findings persuaded us to carry out a similar investigation in Cardiff, and a preliminary report confirming the Newcastle results has already been presented (Pritchard *et al.*, 1972). This paper provides further details of the techniques involved in the MEM test, and reports a number of modifications designed to improve its value as a clinical procedure.

### MATERIALS AND METHODS

Approximately 15 ml of venous blood was collected from healthy hospital staff and from patients with malignant disease. The blood was placed in a siliconized Universal container along with about 40 small (3 mm) glass beads. The silicon-rubber lined cap was replaced and defibrination carried out by continuous inversion until a fibrin clot resulted (usually 5–15 min). The defibrinated blood was pipetted into a 15 ml siliconized centrifuge tube, noting the volume, and centrifuged at 1500 *g* for 10 min in a swing out head. Lymphocytes were harvested either by (a) a methylcellulose technique as used by Hughes and Caspary (1970) and Caspary and Field (1971), or (b) a Ficol-Triosil technique (J. A. Forrester, private communication). For the latter, 9 g of Ficol was dissolved in 100 ml of distilled water; 24 ml of distilled water was added to 20 ml of Triosil-75 (Glaxo) and 40 ml of this diluted

Triosil was then added to 100 ml of the 9% Ficol and the mixture stored in a dark bottle at 4°C. An aliquot of 3 ml of the Ficol-Triosil solution was stored in a siliconized centrifuge tube at 37°C for later use. The buffy coat at the interface of the defibrinated blood after centrifugation was diluted with about twice its volume of medium 199 (Gibco) and this was layered on to the surface of the 3 ml aliquot of Ficol-Triosil, which should not be allowed to stand for longer than 2 min before centrifugation at 1500 *g* for 20 min. The liquid was carefully collected down as far as the interface between medium 199 and Ficol-Triosil and added to 8 ml of medium 199. The lymphocytes from either technique were washed 3 times by centrifugation at 1500 *g* for 10 min and resuspended in medium 199. Permanent slides stained with Jenner Geimsa were made of every preparation for cell counting.

For macrophage production, 20 ml of sterile liquid paraffin BP was injected into the peritoneum of 400–600 g Hartley albino guinea-pigs. Four to 10 days later the animals were exsanguinated and 60 ml of Hanks solution containing 5 units of heparin/ml (preservative free) injected into the peritoneal cavity. The abdomen was gently pummelled and then opened and the peritoneal exudate transferred with a 10 ml pipette into centrifuge tubes. The excess liquid paraffin was pipetted off the top and the macrophages washed once with Hanks/heparin solution and twice with medium 199 and resuspended so that the cell concentration was $10^7$/ml. Each suspension was irradiated to a dose of 150–200 rad from a $^{137}$Caesium source.

The encephalitogenic factor (EF) was prepared from a human brain by the method described by Caspary and Field (1965, 1971). For use, 1·0 mg was dissolved in medium 199 so that the final concentration was 100 $\mu$g/0·1 ml.

*Electrophoretic measurements*

1·0 ml of medium 199 was put in a bijou bottle followed by 0·1 ml of antigen in medium 199, 1·0 ml of the lymphocyte preparation and 1·0 ml of the macrophage suspension. This final volume of 3·1 ml was sufficient to fill the Zeiss Cytopherometer. It is important to keep the pH of all solutions as near to 7·2 as possible. Each sample stood at room temperature (20–23°C) for at least 90 min before being run slowly, taking about 20 sec, into the Zeiss instrument. Cells in focus in the stationary layer were selected and measured over one square of the eyepiece graticule (= 16 $\mu$m) in both directions, and the pairs of times averaged if they did not differ by more than 10%. Such differences are due to several factors: sample turbulence or drifting due to non-uniform temperature; microleaks at taps or joints; cell collisions; small gas bubbles in the test chamber, etc. Usually between 20% and 40% of all timings were rejected for failing to meet the arbitrary 10% standard. It should be emphasized that the intention in the first phase of this investigation was to verify the Newcastle findings, using the techniques described by Field and Caspary. The somewhat empirical methods of data selection and handling were justified partly by this limited intention and partly by the lack of detailed knowledge of all the parameters affecting the test. A more rigorous examination of these parameters must wait for a later phase of the investigation.

A second lymphocyte/macrophage suspension without EF was made up from every sample and measured to give the " control " value for that sample. The constancy of the timings from these controls during each day's run, and from week to week, is a good guide to the state of the apparatus and technique. A retrospective analysis of the results on which this report is based showed that the extreme variation in the timings from control samples without EF over 4 months was surprisingly small at $\pm$ 2·2%. Samples were " scrambled " so that those containing EF and control samples without EF were mixed and presented for blind measurement in a random order, except where the test was part of a concurrent investigation that precluded scrambling. Sets of times from one " normal " and two " malignant " samples are shown in Table I. Unlike the results quoted by Field and Caspary, each individual macrophage timing did not always fall with certainty into the appropriate category of " normal " or " malignant ". The times were therefore recorded in two columns, one containing all pairs which averaged 3·3 sec or less (A in Table I) and the other column containing any pairs that averaged more than 3·3 sec (B in Table I). Measurements on each sample were stopped as soon as one column had accumulated 10 good pairs, and these were then averaged and used as the result

TABLE I.—*Typical Record of Timings from* 3 *Consecutive Samples* (*Times are in Seconds*)

| Sample No. 291 | | | | Sample No. 292 | | | | Sample No. 293 | | | |
|---|---|---|---|---|---|---|---|---|---|---|---|
| No EF | | With EF | | No EF | | With EF | | No EF | | With EF | |
| A | B | A | B | A | B | A | B | A | B | A | B |
| 3·0 | | 3·0 | 3·6 | 3·0 | | | 3·6 | 2·8 | | 3·0 | 3·5 |
| 3·2 | | 3·1 | 3·6 | 2·8 | | | 3·8 | 3·1 | | 3·1 | 3·6 |
| 2·9 | | 2·9 | 3·8 | 3·0 | | | 3·4 | 2·9 | | 3·1 | 3·7 |
| 3·0 | | 2·8 | 3·8 | 3·3 | | | 3·5 | 3·0 | | 3·3 | 4·1 |
| 3·0 | | 3·2 | | 3·1 | | | 3·6 | 3·0 | | | 3·8 |
| 3·1 | | 3·2 | | 3·2 | | | 3·7 | 3·2 | | | 4·2 |
| 3·0 | | 2·8 | | 2·9 | | | 3·5 | 3·3 | | | 3·3 |
| 2·8 | | 2·8 | | 3·1 | | | 3·7 | 3·3 | | | 3·6 |
| 3·0 | | 3·2 | | 2·8 | | | 3·4 | 2·8 | | | 3·4 |
| 3·2 | | 3·1 | | 2·9 | | | 3·4 | 2·8 | | | 3·7 |
| 3·0 | | 3·0 | | 2·9 | | | 3·5 | 2·7 | | | 3·5 |
| 3·3 | | 3·2 | | 3·1 | | | 3·7 | 2·8 | | | 3·8 |
| 2·8 | | 3·0 | | 3·0 | | | 3·5 | 3·2 | | | 3·4 |
| 2·9 | | 3·3 | | 3·3 | | | 3·8 | 3·3 | | | 3·4 |
| 2·9 | | 3·1 | | 3·0 | | | 3·6 | 2·9 | | | 3·5 |
| 3·1 | | 3·2 | | 3·3 | | | 3·8 | 2·8 | | | 3·5 |
| 2·7 | | 2·9 | | 3·0 | | | 3·3 | 2·9 | | | 3·4 |
| 2·9 | | 2·9 | | 3·1 | | | 3·4 | 3·0 | | | 3·5 |
| 2·8 | | 3·3 | | 2·8 | | | 3·4 | 2·9 | | | 3·5 |
| 3·0 | | 3·2 | | 2·8 | | | 3·6 | 3·0 | | | 3·6 |
| 2·98 | | 3·06 | | 3·02 | | | 3·56 | 2·985 | | | 3·60 |
| | | 2·7% | | | | | 17·9% | | | | 20·6% |

from that sample. The inclusion of all of the timings from both columns would not have caused a single false result in our series. Nevertheless, this method of recording gave useful information about the state of the technique from day to day.

Table II shows a retrospective analysis of the numbers of " wrong " pairs in each of the four categories of suspension as the investigation proceeded, expressed as a percentage of the numbers of " right " pairs from successive groups of 10 " malignant " or 5 " normal " samples. The table starts from May 1972 (top line) and covers the 3 months represented by the block of 79 samples which confirmed the Newcastle findings. Columns A and C are from suspensions without EF and therefore, as expected, show no difference between " normal " and " malignant " samples. The fall in the numbers in A, B and C reflects increasing experience with the technique and with the Cytopherometer. The larger numbers in B compared with A and C suggest that a small proportion of macrophages fail to react with the MSF and therefore give " fast " pairs which fall into the " wrong " category for column B. The suspension in column D should show no reaction, and therefore numbers in D should run parallel to numbers in A and C. The large and rather erratic number of slowed cells in column D, not present in A or C and not improving with operator experience, suggests a non-specific EF-stimulated guinea-pig lymphocyte reaction varying from pig to pig (or batch to batch). Since entries in column D

TABLE II.—*Percentage Rate of Pairs of " Wrong " Timings from Groups of* 10 *" Malignant " or* 5 *" Normal " Samples*

| "Malignants" | | "Normals" | |
|---|---|---|---|
| A | B | C | D |
| No EF | With EF | No EF | With EF |
| 13 | 33 | 22 | 16 |
| 10 | 35 | 6 | 2 |
| 12 | 23 | 12 | 18 |
| 5 | 15 | 4 | 28 |
| 0 | 7 | 2 | 14 |

represent " slow " timings which are therefore " wrong " from a " normal " subject, they would be " right " timings if recorded from a " malignant " sample containing EF, and therefore a non-specific reaction of the type postulated would not show up in column B, but would just add to the much more numerous cancer-stimulated slowed cells.

The percentage change in mobility induced by the antigen is 100 (T—C)/C where T is the average migration time for macrophages in the test suspension containing lymphocytes, macrophages and antigen, and C is the average time from the control suspension without antigen. There is no advantage in converting timings into absolute mobilities for this test, so long as measurements are done in one type of apparatus only, with factors such as temperature, voltage or current, pH etc. held constant. All the timings reported here were made over a distance of 16 $\mu$m in medium 199 at pH 7·2 and 23·0±0·05°C, using a stabilized current of 9·5 mA which in our Cytopherometer required a voltage of 200–190 V depending on electrode polarization.

### RESULTS

This investigation was carried out between May 1971 and August 1972. Blood samples from 333 sources were examined with the MEM test. At first it proved extremely difficult to obtain the consistent results claimed by Field and Caspary. We now know that for the first 230 samples a number of adverse factors were operating, often together, making the development of a satisfactory technique both difficult and time-consuming. These problems are outlined briefly in the following points: (a) prior to November 1971 all electrophoretic measurements were made in an apparatus built in this hospital. Although our apparatus was quite capable of accurate and consistent performance in straight electrophoresis, we now know that in its original form it could not give correct results in the MEM test due to the use of platinized platinum electrodes. Subsequent experiments showed that macrophage slowing is lost in the presence of platinized platinum electrodes, although clean unplatinized platinum shows no such effect. This unexpected " poisoning " awaits further investigation. In November 1971 the test was transferred to the Zeiss Cytopherometer in which most of the results reported here were obtained. Since it is not possible to determine retrospectively the extent to which the " poisoning " was operating from day to day, it is difficult to correlate the poor results from the first 230 samples with the " adverse factors " listed here. A detailed account of the early results is therefore of little value and is omitted. (b) In many early blood samples the lymphocyte yields were low (less than $10^5$/ml); improvements in the preparation techniques as the work progressed resulted in substantially increased yields, an important factor for the success of the test, as shown in Table III. (c) The

TABLE III.—*Dependence of Macrophage Percentage Slowing on number of Lymphocytes/ml in Test Suspension*

| Lymphs/ml | Mean time (sec) | % Slowing |
|---|---|---|
| Without EF | | |
| $1·0\times10^5$ | 3·04 | — |
| $1·25\times10^6$ | 3·03 | 0 |
| With 100 $\mu$g EF | | |
| 0 | 3·04 | 0·3 |
| $1·0\times10^5$ | 3·39 | 11·8 |
| $2·5\times10^5$ | 3·43 | 13·3 |
| $5·0\times10^5$ | 3·48 | 14·8 |
| $1·25\times10^6$ | 3·63 | 19·8 |
| $2·5\times10^6$ | 3·75 | 23·8 |

facilities originally available to us prevented effective isolation of the guinea-pigs used in the test. Guinea-pigs are prone to infections from other laboratory animals and from humans, and this sensitizes the animals, which renders the macrophages less effective in the MEM test (Diengdoh and Turk, 1968). Hartley albino pure bred guinea-pigs free from infection were not always available in the early stages of the work, and other unspecified strains were sometimes used until the importance of this factor was realized. The later samples on which this report is based were examined against infection-free Hartley albino pigs from a closed breeding colony or obtained from a single reliable source. (d) Macrophages are easily identified in the Zeiss Cytopherometer because they contain liquid paraffin droplets. However, experience and a period of training seem to be necessary before reproducible results can be obtained. As soon as one operator becomes " familiar " with the somewhat temperamental Cytopherometer there is a tendency to depend on that person for measurements. The urgency of accumulating reliable data precludes the time-consuming investigation that would be needed to assess the real significance of operator-dependent factors. For this reason our electrophoretic measurements were made by one operator (JAVP).

This paper is based on the last 103 consecutive samples measured, starting from May 1972 when the adverse factors outlined above had been effectively eliminated. The first 79 of these samples provided independent confirmation of the MEM test as described by Field and Caspary. These results have been described in detail elsewhere (Pritchard *et al.*, 1972) and in this paper they are condensed (along with the later results) into the histogram of Fig. 1. This shows the clear separation into a " normal " group with percentage slowing not greater than 3% and a " malignant " group with percentage slowing not less than 13%. The absence of overlap is fully confirmed by our results. One sample, thought at the time to be " normal ", gave a percentage slowing of 19% (later repeated as 21%) in the middle of the " malignant " range. On further investigation, the subject was found to have a history of sarcoidosis, one of the few non-malignant conditions reported by Field and Caspary as giving a positive result in the MEM test. Two further sarcoidosis subjects

FIG. 1.—Histogram showing macrophage percentage slowing in the MEM test on 100 blood samples from healthy controls and patients with malignant disease.

FIG. 2.—Dependence of percentage slowing in the MEM test on radiation dose to the guinea-pig macrophage suspension for 4 " malignant " (solid lines) and 1 " normal " (broken line) samples.

were added later, with similar results, to give the small group of 4 positives in the upper section of Fig. 1. Otherwise, all our " normal " subjects were healthy hospital staff. The rest of this paper presents a preliminary account of our early attempts to improve the test towards future clinical use.

With better understanding of the test improvement should be possible under 3 headings: (1) simplification of the electrophoresis apparatus and technique; (2) simplification of the sample handling and preparative procedures; (3) greater separation of the " malignant " and " normal " results. It is to be expected that these 3 aspects are considerably interdependent; for example, an improvement in (3) could be expected to relax the requirements for (2) and (1). Some progress has already been made under (1) but a discussion of apparatus considerations will be presented separately. Under heading (2) the Ficol-Triosil technique of lymphocyte separation already represents a considerable simplification. This technique always produced very good yields of lymphocytes with minimal contamination by polymorphs and red cells, and requires only 60 min of sample preparation time in contrast to the 130 min required by the methylcellulose technique specified by Field and Caspary. We have also found that storage of the separated lymphocytes for 24 hours at 4°C produces no significant change in macrophage percentage slowing, and that storage for 72 hours at 4°C is possible with some reduction in slowing.

Several improvements have been introduced under (3). Percentage slowing can be increased by using a larger concentration of lymphocytes/ml in the reaction mixture, as shown for one typical " malignant " sample in Table III. No corresponding effect was found with " normal " samples. The macrophages used in the first part of this investigation, leading to Fig. 1, were irradiated to a dose of 150–200 rad, as used by Field and Caspary. This dose was sufficient to eliminate most of the mixed lymphocyte interaction, but was not sufficient to achieve maximum slowing, as shown in Fig. 2, in which percentage slowing is

TABLE IV.—*Effect of Radiation Dose and Incubation Regimen on Macrophage Percentage Slowing in the MEM Test*

| | Single stage incubation at: | | | Split incubation: Lymphocytes + EF at 23°C: Supernatant + macrophages at: | | | |
|---|---|---|---|---|---|---|---|
| | 23°C 0 rad A | 23°C 200 rad B | 37°C 200 rad C | 23°C 200 rad D | 37°C 0 rad E | 37°C 200 rad F | 37°C 2500 rad G |
| Sample number | "Malignants" | | | | | | |
| 322 | — | 21·0 | — | 24·0 | — | 40·0 | — |
| 323 | — | 16·8 | 15·0 | 17·0 | — | 27·0 | — |
| 326 | — | 18·4 | 17·0 | 17·9 | — | 30·5 | — |
| 327 | — | 18·3 | 18·9 | 18·2 | — | 32·3 | — |
| 329 | — | 17·3 | — | — | — | 25·8 | — |
| 328 | 1·8 | 16·6 | — | — | 21·4 | 25·1 | 25·8 |
| 330 | 0·5 | 17·2 | — | — | 18·2 | 29·0 | 28·6 |
| 331 | 1·0 | 16·6 | — | — | 18·6 | 22·6 | 22·8 |
| 333 | — | 15·6 | — | — | — | 30·0 | — |
| | "Normals" | | | | | | |
| 325 | — | 0·1 | −0·1 | −1·0 | — | −0·7 | — |
| 332 | 1·8 | 2·0 | — | — | 0·2 | 1·3 | 1·8 |

plotted against radiation dose for 4 "malignant" and 1 "normal" samples.

The use of high radiation doses could hardly be described as a simplification of the test, and therefore alternative methods were sought for eliminating the mixed lymphocyte interaction completely. Incubation was split into two stages: first, the patient's lymphocytes were mixed with EF and incubated for 90 min at either 23°C or 37°C to stimulate the release of MSF; lymphocytes were then removed from this suspension by centrifugation and the cell-free supernatant was added to the macrophage suspension for a second 90 min incubation at 23°C or 37°C. In this way, human lymphocytes did not come into contact with the guinea-pig lymphocytes present in the macrophage suspension, and therefore a mixed lymphocyte interaction should not occur, thus removing the need for prior irradiation. Table IV presents the combined results of a number of experiments of this type, from which the following conclusions emerge, some of which require further investigation: (1) column B represents the result of the test as developed by Field and Caspary, and is now confirmed by our results. (2) There is no benefit in raising the temperature of the complete mixture from 23°C to 37°C during single stage incubation (columns C/B). (3) Results are unaffected when incubation at 23°C is split into two stages, showing that MSF is a soluble factor released by the interaction of sensitized lymphocytes with EF (columns D/B). (4) When human and guinea-pig lymphocytes are kept apart by split incubation, mixed lymphocyte interaction is no longer a problem, and irradiation of the macrophage suspension can be omitted without loss of result (columns E/B). (5) A large improvement in the MEM test is obtained when macrophages which were previously irradiated to at least 200 rad are incubated for 90 min at 37°C with cell-free supernatant containing MSF (columns F/B). (6) Using the incubation regimen just described, no further improvement is seen with doses larger than 200 rad (columns G/F). (7) The results from "normal" subjects are unaffected by these changes in irradiation and incubation.

### DISCUSSION

This investigation has confirmed the results of Field and Caspary (1970) and Caspary and Field (1971) and verifies the MEM test as an *in vitro* laboratory technique for the detection of malignant disease. All patients with malignant disease in our series gave macrophage percentage slowing between 13% and 29%, while all healthy controls (with the exception of 3 sarcoidosis subjects) were below 3·4% (Fig. 1), thus emphasizing again the absence of overlap between " malignant " and " normal " subjects. The absence of overlap to date suggests that it may prove difficult to investigate the rise in sensitization during early neoplastic involvement, since subjects may pass quickly through this stage to a full positive MEM response. We have not found the sensitization of laboratory staff to EF reported from Newcastle, but this may come in the future with increased exposure to the antigen.

Table III shows the importance of maintaining a high concentration of lymphocytes/ml. Fig. 1 and Table IV were obtained with numbers in the range $1 \times 10^6$/ml–$5 \times 10^6$/ml, at which percentage slowing is approaching a maximum. In addition to maintaining the necessary high lymphocyte yield and requiring a shorter preparation time, the Ficol-Triosil method adopted by us as standard, gave minimal contamination by polymorphs and red cells, an important factor when training an inexperienced operator. We have found that " wrong " pairs of timings were more numerous on days when the macrophage suspension was contaminated with red cells, although the reason for this is not clear.

The split incubation technique described above increases percentage slowing from " malignant " samples into the range 22%–40% without any apparent change in the result from " normal " subjects, as shown in Table IV. Table IV provides a direct comparison between the test as described by Field and Caspary (column B) and the Cardiff-modified MEM test of column F, which has now been adopted as our standard procedure and as a basis for further simplification and development. This change in sample handling has greatly increased the already wide gap between " malignant " and " normal " subjects, which is an important step towards possible clinical use of the MEM test. This larger difference reduces the possibility of false results, is more easily seen by an inexperienced operator, and may lead to simpler techniques for greater convenience in putting the test into practical use. For example, using the incubation regimen of column E in Table IV it is possible to dispense with irradiation while still retaining a percentage slowing slightly higher than that provided by the original Newcastle methods. It is not yet clear why irradiation of the macrophage suspension to 200 rad should increase the percentage slowing to the values in column F of Table IV, since mixed lymphocyte interaction should be absent, and this requires further investigation.

Results are not yet available in our series from a population of " normals " with a wide variety of non-malignant illnesses, and much laboratory work remains to be done before the MEM test can be used as a routine clinical procedure. Even with simplification of the techniques, the test will have limited value as a screening procedure unless the patient has specific localizing signs or symptoms, since there is at present no simple way to locate early malignant disease, except for a few sites such as cancer of the cervix. In view of this we are now applying the MEM test to situations where it can be used in conjunction with existing clinical and ancillary investigations; this includes an evaluation of its usefulness for diagnosing malignancy in female breast swellings, the results of which we hope to publish in the near future.

We are grateful to our radiotherapy colleagues for advice and discussion, and we particularly thank Professor E. J. Field and E. A. Caspary of the Medical Research Council Unit for Demyelinating Diseases, Newcastle upon Tyne. To the staff of the Tenovus Laboratory and the Animal House we also extend our thanks. We are extremely grateful to Tenovus, the South Wales and Monmouthshire Cancer Research Council and the Department of Health and Social Security for financing this project.

### REFERENCES

CASPARY, E. A. & FIELD, E. J. (1965) An Encephalitogenic Protein of Human Origin; Some Chemical and Biological Properties. *Ann N.Y. Acad. Sci.*, **122**, 182.

CASPARY, E. A. & FIELD, E. J. (1971) Specific Lymphocyte Sensitisation in Cancer: is there a Common Antigen in Human Malignant Neoplasia? *Br. med. J.*, ii, 613.

DIENGDOH, J. V. & TURK, J. L. (1968) Electrophoretic Mobility of Guinea Pig Peritoneal Exudate Cells in Hypersensitivity Reactions. *Int. Archs Allergy*, **43**, 297.

FIELD, E. J. & CASPARY, E. A. (1970) Lymphocyte Sensitisation: an *in vitro* Test for Cancer? *Lancet*, ii, 1337.

FIELD, E. J. & CASPARY, E. A. (1972) Lymphocyte Sensitisation in Advanced Malignant Disease: a Study of Serum Lymphocyte Depressive Factor. *Br. J. Cancer*, **26**, 164.

HUGHES, D. & CASPARY, E. A. (1970) Lymphocyte Transformation *in vitro* Measured by Tritiated Thymidine Uptake. *Int. Archs Allergy*, **37**, 506.

PRITCHARD, J. A. V., MOORE, J. L., SUTHERLAND, W. H. & JOSLIN, C. A. F. (1972) The Macrophage Electrophoretic Mobility (MEM) Test for Malignant Disease: an Independent Confirmation. *Lancet*, ii, 627.